# Monocyte-to-HDL Cholesterol Ratio and Complete Blood Count-Derived Inflammatory Markers in Myasthenia Gravis: A Cross-Sectional Case–Control Study

**DOI:** 10.3390/jcm15218738

**Published:** 2026-11-09

**Authors:** Burak Özaltun, Merve Hilal Ceren Akgör

**Affiliations:** Department of Neurology, Ankara Atatürk Sanatorium Training and Research Hospital, 06290 Ankara, Turkey; cerenmervee@gmail.com

**Keywords:** myasthenia gravis, monocyte-to-HDL cholesterol ratio, neutrophil-to-lymphocyte ratio, systemic inflammation response index, systemic inflammation

## Abstract

**Background/Objectives:** We evaluated the monocyte-to-high-density lipoprotein (HDL) cholesterol ratio (MHR) and complete blood count (CBC)-derived inflammatory ratios in myasthenia gravis (MG). **Methods:** In this cross-sectional case–control study, 80 patients with MG and 87 controls were compared. MHR, neutrophil-to-lymphocyte ratio (NLR), platelet-to-lymphocyte ratio (PLR), lymphocyte-to-monocyte ratio (LMR), and systemic inflammation response index (SIRI) were analyzed with Mann–Whitney U tests, ROC analysis, and multivariable logistic regression with covariates selected using a directed acyclic graph. **Results:** MHR was higher in MG (median 0.0091 vs. 0.0058; *q* < 0.001), as were NLR, PLR, and SIRI, whereas LMR was lower. AUCs were 0.779 (SIRI), 0.774 (LMR), and 0.700 (MHR). Each 0.001-unit increase in MHR was associated with 1.23-fold higher odds of MG (95% confidence interval 1.11–1.38; *p* < 0.001), and adding MHR to the covariates increased the model area under the curve from 0.752 to 0.823 (*p* = 0.017; complete-case analysis, *p* = 0.216), mainly through the monocyte count. No marker correlated with severity (all *p* > 0.12). **Conclusions:** MHR and CBC-derived ratios differed between patients with MG and controls independent of demographic and metabolic covariates, largely reflecting monocyte counts, but were not associated with severity and do not appear suitable for disease monitoring.

## 1. Introduction

Myasthenia gravis (MG) is an autoimmune disease characterized by autoantibodies against components of the postsynaptic membrane at the neuromuscular junction and presents with muscle weakness and fatigability. Antibodies against the acetylcholine receptor (AChR) are detected in most cases, whereas some patients have antibodies against muscle-specific tyrosine kinase (MuSK) or low-density lipoprotein receptor-related protein 4 (LRP4); approximately 10% of patients are seronegative [1,2,3].

Although B cells and autoantibodies play a central role in the pathogenesis of MG, the contribution of the innate immune system and the systemic inflammatory response to disease onset and course has been increasingly emphasized in recent years. Monocytes contribute to autoimmune processes through antigen presentation, direction of T-cell differentiation, and production of proinflammatory cytokines. Conversely, high-density lipoprotein (HDL) enhances cholesterol efflux from the monocyte membrane via the ABCA1 and ABCG1 transporters; this disrupts the structure of cholesterol-rich membrane microdomains (lipid rafts) in which Toll-like receptors cluster, thereby limiting proinflammatory signaling and monocyte activation [4].

The monocyte-to-HDL cholesterol ratio (MHR), which expresses the ratio between these two opposing mechanisms, summarizes the balance between inflammatory and anti-inflammatory activity in a single number and can be calculated from routine tests at no additional cost. MHR has been investigated in cardiovascular diseases, metabolic syndrome, and various autoimmune conditions and has been associated with disease presence, cardiometabolic risk, or prognosis [5,6,7]. Similarly, the neutrophil-to-lymphocyte ratio (NLR), platelet-to-lymphocyte ratio (PLR), lymphocyte-to-monocyte ratio (LMR), and systemic inflammation response index (SIRI) are markers derived from the complete blood count (CBC) that are regarded as reflecting systemic inflammation [8,9,10,11].

Although data on these markers in MG are accumulating, most available studies have been limited to NLR, PLR, and SIRI and have focused mainly on disease severity or the risk of crisis [8,9,10,11,12,13]. In MG, MHR has been measured only as part of a broad marker panel; it was not addressed as a primary outcome and did not differ significantly between groups in that single study [8]. In contrast, MHR has been associated with disease presence and severity in neuromyelitis optica spectrum disorder, Guillain–Barré syndrome, and parkinsonian syndromes [7,14,15]. In this study, we aimed to compare MHR and CBC-derived inflammatory ratios between patients with MG and healthy controls, to determine their discriminative performance, and to evaluate their associations with indicators of clinical severity. To our knowledge, this is the first study to examine MHR as a primary marker in MG and to test the robustness of the findings using multiple-comparison correction and multivariable modeling.

## 2. Materials and Methods

### 2.1. Study Population

We retrospectively reviewed 194 patients with myasthenia gravis (MG) followed at the Neurology Clinic of Ankara Atatürk Sanatorium Training and Research Hospital between January 2016 and June 2026. The diagnosis was confirmed by typical clinical findings together with electrophysiological testing (repetitive nerve stimulation and/or single-fiber electromyography) and/or serological test results. The control group was assembled retrospectively from the records of individuals who attended the check-up outpatient clinic of our hospital during the study period for routine health assessment or health reports; several thousand such individuals are examined each year, but the exact number for the study period could not be retrieved. To obtain an age and sex distribution similar to that of the MG group, laboratory records of individuals in the corresponding age and sex strata were reviewed, and those without known disease, diabetes, or regular medication (in particular lipid-lowering therapy) who had a CBC and lipid profile from the same visit were included; because eligible candidates were scarce in the older age strata, hypertension alone was not an exclusion criterion for controls. Controls were thus frequency-matched to the patients for age and sex at the group level; no individual matching and no consecutive or random sampling was performed. The study protocol was approved by the Scientific Studies Ethics Committee of Ankara Atatürk Sanatorium Training and Research Hospital, University of Health Sciences (decision no: 2024-BÇEK/686, date: 9 September 2026), and this study was conducted in accordance with the principles of the Declaration of Helsinki.

### 2.2. Exclusion Criteria

To minimize confounding, we applied strict exclusion criteria: (1) age under 18 years; (2) concomitant autoimmune or neuroinflammatory disorders; (3) active infection (defined by fever, radiological evidence, or C-reactive protein [CRP] > 10 mg/L unrelated to MG); (4) malignancy; (5) severe hepatic or renal dysfunction; (6) acute cerebrovascular events; and (7) missing laboratory data required to calculate the inflammatory ratios (patients without a CBC or lipid profile). The same exclusion criteria were applied to the control group.

Of the 194 patients with MG screened, 114 were excluded on the basis of these criteria: concomitant autoimmune or neuroinflammatory disease (*n* = 25), active infection (*n* = 23), malignancy (*n* = 11), severe hepatic or renal dysfunction (*n* = 7), acute cerebrovascular event (*n* = 5), age under 18 years (*n* = 4), and missing laboratory data, i.e., no CBC or lipid profile available (*n* = 39). Thus, 80 patients with MG were included in the analysis (Figure 1). Excluded patients were younger than those included (median age at sampling 47 (IQR 33–63) vs. 64 (IQR 53–74) years; *p* < 0.001), partly because the exclusions included children and patients with other autoimmune diseases, whereas the proportion of women was similar (55.3% vs. 60.0%; *p* = 0.512); other clinical characteristics were not consistently available for excluded patients. Four patients with MG had a CRP level above 10 mg/L (12–16 mg/L): two were sampled during a myasthenic exacerbation and two shortly after thymectomy. In all four, procalcitonin, cultures, chest imaging, and infectious-disease consultation showed no active infection; the elevation was attributed to these MG-related conditions, and the patients were retained. Because the two controls with CRP above 10 mg/L had no such explanation and were excluded, a sensitivity analysis excluding these four patients is reported in Section 3.7. Of the 135 candidate controls in the relevant age and sex strata who were assessed for eligibility, 48 were excluded (no lipid profile, *n* = 43; unusable clotted sample, *n* = 3; CRP above 10 mg/L, *n* = 2), and 87 constituted the control group.

### 2.3. Data Collection

Demographic characteristics (age, sex, and smoking), comorbidities (diabetes, hypertension, and use of lipid-lowering therapy), and MG-specific clinical data (disease duration, MG type, antibody status, Myasthenia Gravis Foundation of America [MGFA] clinical classification, Myasthenia Gravis Activities of Daily Living [MG-ADL] score, history of myasthenic crisis, thymectomy and thymic histopathology, and treatments received) were retrospectively obtained from patient files and the electronic medical record system. Treatment at the time of sampling was defined as the medications in use on the date of blood sampling, as recorded in the medical records. Patients receiving corticosteroids and/or immunosuppressants (azathioprine, rituximab, or eculizumab) were classified as treated, and those receiving pyridostigmine alone or no treatment as untreated. MGFA class and MG-ADL score were those recorded at the visit at which the blood sample was obtained. Antibody status was classified as unknown when testing had been performed at another center before referral, and the result could not be retrieved.

### 2.4. Laboratory Measurements

CBC and lipid profile results were retrospectively retrieved from the hospital laboratory information system. All measurements had been performed in the central hematology and biochemistry laboratories of our hospital using the same automated analyzers (Sysmex Corporation, Kobe, Japan) and routine standard methods throughout the study period. For patients with MG, the first sample obtained at an outpatient visit or hospital admission was used; for controls, the sample obtained during routine health screening was used. In accordance with institutional practice, lipid measurements are performed on fasting samples; however, because the duration of fasting could not be independently verified from the records, this is acknowledged among the limitations of this study. The inflammatory ratios were calculated using the following formulas:

MHR = monocyte count (10^3^/µL)/HDL cholesterol (mg/dL);
NLR = neutrophil count/lymphocyte count;
PLR = platelet count/lymphocyte count;
LMR = lymphocyte count/monocyte count;
SIRI = (neutrophil count × monocyte count)/lymphocyte count.

Because unit conventions for MHR vary in the literature, the units used in this study are stated explicitly, and the cut-off value was derived from our own data by ROC analysis rather than adopted from external sources.

### 2.5. Statistical Analysis

Descriptive and univariable analyses were performed using IBM SPSS Statistics 22 (IBM Corp., Armonk, NY, USA); multivariable analyses were performed in R version 4.3.3 (R Foundation for Statistical Computing, Vienna, Austria) using the mice (version 3.16.0), pROC (version 1.18.5), and rms (version 6.7.1) packages. The normality of continuous variables was assessed using the Shapiro–Wilk test. Because most variables, including the inflammatory ratios and hematological parameters, were not normally distributed, continuous variables are presented as median (interquartile range [IQR]) and were compared between groups using the Mann–Whitney U test. Because LDL and total cholesterol were normally distributed, the independent-samples *t*-test was additionally applied to these variables for confirmation, and the results were unchanged. Categorical variables are presented as *n* (%) and were compared using the chi-square test or, where appropriate, Fisher’s exact test. To control type I error inflation arising from the simultaneous comparison of multiple inflammatory markers, Benjamini–Hochberg false discovery rate (FDR) correction was applied to the 15 between-group comparisons of laboratory parameters and inflammatory ratios, which were prespecified as a single family of tests; adjusted *q* values are reported.

The ability of the inflammatory ratios to discriminate patients with MG from controls was evaluated using receiver operating characteristic (ROC) analysis; the area under the curve (AUC) and its 95% confidence interval (CI) were calculated. Optimal cut-off values were determined by maximizing the Youden index (sensitivity + specificity − 1). For LMR, lower values were considered indicative of disease. AUCs were compared using the DeLong test.

Binary logistic regression was used to evaluate the independent association of each marker with MG. Covariates were selected a priori with a directed acyclic graph (DAG) built from published evidence (Figure 2) [5,16,17,18,19,20,21,22,23,24,25,26,27,28]. In the DAG, age, sex and smoking are common causes of MG status and of the inflammatory indices and therefore form the minimal sufficient adjustment set for confounding (Model 1). Because controls were selected from individuals without known disease attending routine health screening, inclusion was conditioned on health status; this selection opens non-causal paths between MG status and the markers through hypertension, BMI, diabetes and lipid-lowering therapy [27]. To block these paths, and in keeping with the observed between-group differences, BMI and hypertension were added in Model 2, which was prespecified as the primary model. Diabetes and lipid-lowering therapy could not be included because no control participant had either condition (violation of positivity); their influence was addressed by restriction, re-fitting Model 2 after excluding patients with MG who had diabetes or used lipid-lowering therapy. Because BMI may also lie on the pathway from MG through corticosteroid treatment to the markers [22], Models 1 and 2 are reported side by side.

BMI was missing for 66 of 167 participants (39.5%; 37 controls and 29 patients). Assuming data were missing at random, missing values were imputed by multivariate imputation by chained equations (predictive mean matching; 40 imputed datasets; 20 iterations). The imputation model included group, age, sex, smoking, BMI, hypertension, diabetes, lipid-lowering therapy, year of sampling, HDL cholesterol, triglycerides, log-transformed CRP and the absolute neutrophil, lymphocyte, monocyte and platelet counts. Estimates were combined with Rubin’s rules. As a sensitivity analysis, Model 2 was re-fitted in participants with recorded BMI (complete-case analysis; *n* = 101; Supplementary Table S1). One patient with missing HDL cholesterol was excluded from the MHR models.

A separate model was fitted for each marker because the indices share their constituent cell counts. MHR was multiplied by 1000 so that odds ratios (ORs) are expressed per 0.001-unit increase. Linearity of each marker in the logit was examined by comparing a linear term with a natural cubic spline (3 degrees of freedom) using likelihood-ratio tests in each imputed dataset (Supplementary Table S2). MHR and SIRI met the linearity assumption. LMR did not on its original scale (*p* = 0.002 in Model 1) but did after log2 transformation and was therefore entered as log2(LMR), with ORs expressed per doubling. NLR showed a non-linear association (median *p* = 0.005) that was not removed by log transformation; it was retained on its original scale and interpreted with caution. With 80 events and 7 model parameters, there were at least 11 events per parameter.

Discrimination of each multivariable model was assessed with the area under the ROC curve (AUC) of the model-predicted probabilities. AUCs and their DeLong variances were pooled across imputations with Rubin’s rules. The added value of each marker was quantified as the difference in AUC between Model 2 and a model containing only the covariates (paired DeLong test). Overall fit was summarized by −2 log-likelihood, the Akaike information criterion (AIC), Cox–Snell and Nagelkerke R^2^, the Brier score and the proportion correctly classified at a predicted probability of 0.5. Calibration was assessed with the Hosmer–Lemeshow test (10 groups; median *p* across imputations), calibration plots, and the calibration slope and intercept. Internal validation used 200 bootstrap resamples in each imputed dataset to obtain optimism-corrected C-statistics and calibration slopes.

To examine whether MHR carried information beyond its two components, Model 2 was re-fitted with the monocyte count and HDL cholesterol entered separately, alone and together, and with MHR added to the model containing both. Nested models were compared with likelihood-ratio tests (median *p* across imputations) and AUCs with paired DeLong tests. Because log(MHR) equals log(monocyte count) minus log(HDL), the ratio constrains the coefficients of these two terms to be equal and opposite; this constraint was tested by comparing a model containing log(MHR) with a model containing log(monocyte count) and log(HDL) as separate terms. Finally, MHR was added to a model containing the covariates and log-transformed CRP to assess whether it contributed information beyond a conventional inflammatory marker.

Within the MG group, associations of the inflammatory ratios with MGFA class, MG-ADL score, and disease duration were examined using Spearman’s rank correlation coefficient. As sensitivity analyses, (i) patients with MG without diabetes or lipid-lowering therapy, (ii) patients who were not receiving corticosteroids or immunosuppressants at the time of sampling, and (iii) patients with CRP ≤ 10 mg/L were each compared separately with controls; in subgroups (ii) and (iii), Model 2 was also re-fitted, and treated and untreated patients were compared. Statistical significance was set at a two-sided *p* < 0.05. MHR was predefined as the primary outcome marker, and analyses of the other ratios were considered exploratory.

## 3. Results

### 3.1. Study Population

A total of 167 participants were included: 80 patients with MG and 87 healthy controls (Figure 1). The groups were similar with respect to age (median 64 (IQR 53–74) vs. 62 (IQR 44–78) years; *p* = 0.500) and sex distribution (proportion of women, 60.0% vs. 58.6%; χ^2^ = 0.033, *p* = 0.856). Smoking status did not differ significantly between the groups (*p* = 0.165). Body mass index (BMI) was higher in the MG group (median 29.4 vs. 25.6 kg/m^2^; *p* = 0.003). Demographic characteristics are presented in Table 1. The prevalence of hypertension was significantly higher in the MG group than in controls (55.0% vs. 17.2%; χ^2^ = 26.01, *p* < 0.001).

The median disease duration in patients with MG was 53.9 months (IQR 18.9–104.3). Overall, 78.8% of patients had generalized MG and 21.3% had ocular MG. Antibody status was AChR-positive in 25 patients (31.6%) and seronegative in 20 patients (25.3%); no MuSK-positive patient was included. Antibody results were unavailable for 34 patients (43.0%) because testing had been performed at other centers before referral and the results could not be retrieved. According to the MGFA classification, 78.8% of patients were in the mild group (Classes I–III) and 21.3% in the severe group (Classes IV–V). The median MG-ADL score was 6 (IQR 2–10). Eighteen patients (22.8%) had undergone thymectomy; histopathology showed thymoma in thirteen and thymic hyperplasia in five. Forty-one patients (51.3%) had a history of myasthenic crisis, and eight patients (10.1%) had refractory disease. At the time of sampling, 73 patients (91.3%) were receiving pyridostigmine, and 44 (55.0%) were receiving corticosteroids or immunosuppressants (corticosteroids, *n* = 14; azathioprine, *n* = 30; rituximab or eculizumab, *n* = 2); the remaining 36 patients (45.0%) were receiving pyridostigmine alone or no treatment. Clinical characteristics are summarized in Table 2.

### 3.2. Laboratory Findings and Inflammatory Ratios

Compared with controls, the MG group had significantly higher leukocyte (*p* = 0.004), neutrophil (*p* < 0.001), and monocyte (*p* < 0.001) counts, higher triglyceride (*p* = 0.008) and CRP (*p* < 0.001) levels, and significantly lower lymphocyte counts (*p* < 0.001) and HDL cholesterol levels (*p* = 0.003). No between-group differences were found in platelet count (*p* = 0.515), LDL (*p* = 0.521), or total cholesterol (*p* = 0.830). After Benjamini–Hochberg correction across the 15 comparisons in Table 3, all of these differences remained significant (all *q* < 0.02), whereas platelet count, LDL, and total cholesterol remained non-significant (all *q* > 0.5) (Table 3).

All five inflammatory ratios differed significantly between the groups, and all of these differences persisted after FDR correction. MHR was markedly higher in the MG group than in controls (median 0.0091 (IQR 0.0062–0.0126) vs. 0.0058 (IQR 0.0046–0.0085); Z = −4.446, *p* < 0.001; *q* < 0.001). Similarly, NLR (2.58 vs. 1.81; *p* < 0.001; *q* < 0.001), SIRI (1.12 vs. 0.62; *p* < 0.001; *q* < 0.001) and PLR (147.1 vs. 119.7; *p* = 0.022; *q* = 0.027) were higher in the MG group, whereas LMR was lower (3.94 vs. 5.79; *p* < 0.001; *q* < 0.001). In univariable comparisons, both components of MHR differed: the monocyte count was higher, and the HDL level was lower in the MG group (monocytes, *p* < 0.001; HDL, *p* = 0.003); in multivariable analysis, however, only the monocyte count remained independently associated with MG (Section 3.5).

### 3.3. Discriminative Performance

In ROC analysis, the highest discriminative ability was achieved by SIRI (AUC 0.779; 95% CI 0.707–0.850) and LMR (AUC 0.774; 95% CI 0.703–0.845). The AUC for MHR was 0.700 (95% CI 0.619–0.781; *p* < 0.001); at a cut-off of 0.0075 determined by the Youden index, sensitivity was 65.8%, and specificity was 72.4%. PLR showed the lowest discriminative ability (AUC 0.603; *p* = 0.022). In pairwise comparisons using the DeLong test, the AUC of MHR did not differ significantly from those of SIRI (*p* = 0.066) or LMR (*p* = 0.103) (Table 4).

### 3.4. Multivariable Analysis

In Model 1, adjusted for age, sex, and smoking, each 0.001-unit increase in MHR was associated with 1.24-fold higher odds of MG (95% CI 1.12–1.36; *p* < 0.001). Further adjustment for BMI and hypertension (Model 2, primary) did not materially change this estimate (OR 1.23, 95% CI 1.11–1.38; *p* < 0.001) (Table 5). The association persisted in the complete-case analysis (OR 1.22, 95% CI 1.04–1.42; *p* = 0.012; *n* = 101) and after excluding patients with diabetes or lipid-lowering therapy (OR 1.20, 95% CI 1.07–1.35; *p* = 0.002). LMR (OR per doubling 0.19, 95% CI 0.09–0.38) and SIRI (OR 5.86, 95% CI 2.63–13.07) were also independently associated with MG in all analyses. NLR was associated with MG in Model 2 (OR 1.85, 95% CI 1.28–2.68; *p* = 0.001) but not in the complete-case analysis (OR 1.38, 95% CI 0.99–1.93; *p* = 0.059), and its relation with MG was non-linear. In Model 2, hypertension was associated with MG for every marker (OR 6.39–9.00), whereas BMI was not (OR 1.01–1.03). In the complete-case sample, BMI was associated with MG when entered alone (OR per kg/m^2^ 1.12, 95% CI 1.04–1.22; *p* = 0.004), but not after adjustment for age, sex and smoking (OR 1.07, 0.99–1.18; *p* = 0.104) or, additionally, hypertension (OR 1.02, 0.91–1.15; *p* = 0.683); the univariable BMI difference was therefore largely accounted for by the higher prevalence of hypertension and the older age of the patients. Adding MHR to the covariates-only model increased the AUC from 0.752 to 0.823 (ΔAUC 0.070, 95% CI 0.012–0.128; *p* = 0.017; Table 6, Figure 3). After internal validation, the optimism-corrected C-statistic was 0.794 and the calibration slope 0.85, indicating modest optimism. All models showed adequate calibration (median Hosmer–Lemeshow *p* 0.522–0.737; Figure 4). In the complete-case subgroup, which did not include the oldest controls, the covariates alone already reached an AUC of 0.876, and the increase from adding MHR was not significant (AUC 0.905; *p* = 0.216).

### 3.5. Contribution of the MHR Components

To examine whether MHR carried information beyond its components, the monocyte count and HDL cholesterol were entered into Model 2 separately (Table 7). The monocyte count alone was strongly associated with MG (OR per 0.1 × 10^3^/µL 1.81, 95% CI 1.35–2.43; *p* < 0.001) and yielded an AUC of 0.826, similar to that of the MHR model (0.823; *p* = 0.753). HDL cholesterol was not independently associated with MG after adjustment for the covariates (OR per 10 mg/dL 0.79, 95% CI 0.59–1.06; *p* = 0.118) and added little discrimination (AUC 0.767; *p* = 0.354 vs. covariates only). When the monocyte count and HDL were entered together, adding MHR did not improve the model (likelihood-ratio *p* = 0.726). The equal-and-opposite constraint implied by the ratio was not rejected (*p* = 0.105), but the coefficient for log(monocyte count) was about four times larger in magnitude than that for log(HDL) (2.41 vs. −0.59). In univariable ROC analysis, the AUC of MHR (0.700) did not differ from that of the monocyte count (0.708; *p* = 0.744) but was higher than that of HDL (0.632; *p* = 0.031). In contrast, MHR remained associated with MG when added to a model containing the covariates and log-transformed CRP (OR 1.21, 95% CI 1.08–1.35; *p* = 0.001) and increased its AUC from 0.811 to 0.850 (ΔAUC 0.039, 95% CI 0.001–0.077; *p* = 0.044).

### 3.6. Association with Clinical Severity

In Spearman correlation analysis within the MG group, no significant association was found between any inflammatory ratio and MGFA class (all |ρ| < 0.18; *p* > 0.12) or MG-ADL score (all |ρ| < 0.18; *p* > 0.12) (Table 8). Likewise, no marker differed between the mild (MGFA I–III, *n* = 63) and severe (MGFA IV–V, *n* = 17) groups (all *p* > 0.15), and no marker correlated significantly with disease duration (all *p* > 0.05).

Values are Spearman’s ρ (*p*). The last column shows the comparison of the MGFA mild (I–III, *n* = 63) and severe (IV–V, *n* = 17) groups using the Mann–Whitney U test. The correlation between MGFA class and MG-ADL score was ρ = 0.857 (*p* < 0.001).

The strong correlation between MGFA class and MG-ADL score (ρ = 0.857; *p* < 0.001) supported the internal consistency of the clinical assessments.

### 3.7. Sensitivity Analyses

Patients with active infection had been excluded from the entire analytic sample (Section 2.2). When patients with MG without diabetes or lipid-lowering therapy (*n* = 53; 27 patients excluded: 18 with diabetes and 12 with lipid-lowering therapy, 3 of whom had both) were compared with controls, the difference in MHR persisted (median 0.0079 vs. 0.0058; *p* < 0.001). In this subgroup, the differences in LMR (*p* < 0.001), SIRI (*p* < 0.001), and NLR (*p* = 0.015) also persisted, whereas the difference in PLR lost significance (*p* = 0.068). At the time of sampling, 44 patients (55.0%) were receiving corticosteroids or immunosuppressants. In the 36 patients who were not receiving these drugs, MHR, NLR, LMR, and SIRI still differed from controls (all *p* ≤ 0.005), whereas PLR did not (*p* = 0.359), and MHR remained independently associated with MG in Model 2 (OR 1.25, 95% CI 1.08–1.44; *p* = 0.003). None of the markers differed between treated and untreated patients (all *p* > 0.13). After excluding the four patients with CRP above 10 mg/L (*n* = 76), all marker differences persisted (*p* ≤ 0.019), and the Model 2 estimate for MHR was unchanged (OR 1.24, 95% CI 1.11–1.39; *p* < 0.001; ΔAUC 0.066, *p* = 0.022). Within the MG group, MHR did not differ significantly between patients receiving (*n* = 12) and not receiving (*n* = 68) lipid-lowering therapy (*p* = 0.107). The sensitivity analyses are presented in Table 9.

Values are median (interquartile range); Mann–Whitney U test versus controls. Patients with active infection were excluded from the entire analytic sample. Treatment status refers to medications in use on the date of blood sampling (Section 2.3). In patients not receiving corticosteroids or immunosuppressants, Model 2 ORs (95% CI) were 1.25 (1.08–1.44) for MHR, 1.77 (1.10–2.87) for NLR, 0.23 (0.09–0.58) per doubling of LMR, and 5.52 (2.11–14.47) for SIRI (*n* = 123). Treated (*n* = 44) and untreated (*n* = 36) patients did not differ in any marker (all *p* > 0.13). After excluding the four patients with CRP > 10 mg/L, Model 2 ORs (95% CI) were 1.24 (1.11–1.39) for MHR, 1.87 (1.26–2.76) for NLR, 0.17 (0.08–0.37) per doubling of LMR, and 6.25 (2.72–14.36) for SIRI (*n* = 162–163). Within the MG group, MHR did not differ between patients receiving (*n* = 12) and not receiving (*n* = 68) lipid-lowering therapy (*p* = 0.107).

## 4. Discussion

In this study, MHR was significantly higher in patients with MG than in healthy controls, and the association remained significant after adjustment for age, sex, smoking, BMI, and hypertension. The higher MHR reflected both a higher monocyte count and a lower HDL level in the MG group, but its discriminative information was carried mainly by the monocyte count. Other inflammatory indices also differed between groups, with higher NLR and SIRI and lower LMR, whereas PLR showed a weaker difference. In contrast, none of the evaluated inflammatory ratios was associated with MGFA class or MG-ADL score. These findings distinguish an association with MG status from an association with clinical severity and suggest that MHR, largely through its monocyte component, reflects a systemic inflammatory difference between patients with MG and controls, without establishing a role in disease monitoring or diagnosis.

The MHR finding is of particular interest because its two components represent biologically distinct but interacting pathways. Monocytes participate in antigen presentation and proinflammatory cytokine signaling, whereas HDL has several immunomodulatory properties, including effects on lipopolysaccharide binding, cholesterol efflux, and monocyte activation [16]. Accordingly, an increased MHR may reflect the balance between circulating monocyte-related inflammatory activity and HDL-associated anti-inflammatory mechanisms. Nevertheless, because the present study was cross-sectional, the observed combination of higher monocyte counts and lower HDL levels cannot establish a mechanistic relationship with MG and may also be influenced by metabolic factors, comorbidities, treatment exposure, or other unmeasured characteristics. In our data, moreover, the discriminative information of MHR was carried almost entirely by the monocyte count: a model containing the monocyte count alone discriminated and the MHR model; MHR added no information once the monocyte count and HDL were entered separately, and HDL was not independently associated with MG after adjustment for BMI and hypertension (Table 7). The ratio should therefore not be taken as evidence that the balance between monocytes and HDL, rather than the monocyte count itself, is altered in MG.

The principal finding differs from that of Jin et al. [8], who reported no significant difference in MHR between patients with MG and controls (0.29 vs. 0.31; *p* = 0.288). Differences in monocyte behavior between the two cohorts provide one plausible explanation. In the study by Jin et al., monocyte counts were identical in the MG and control groups (0.40 × 10^9^/L in both; *p* = 0.957), whereas in our cohort the monocyte count was higher in patients with MG (0.45 vs. 0.34 × 10^3^/µL; *p* < 0.001). In our population, the monocyte difference accounted for most of the separation of the groups by MHR, whereas HDL contributed little independent information (Table 7). However, differences in monocyte counts alone should not be assumed to explain the discordant results. Variation in disease activity, treatment status, demographic and metabolic characteristics, comorbidities, infection, BMI, lipid profiles, and blood-sampling conditions may all influence MHR. Yang et al. also reported that HDL did not differ across NLR tertiles (*p* = 0.770) and was not associated with disease severity (OR 0.416; *p* = 0.196) [12], further suggesting that lipid abnormalities may not be uniform across MG populations.

Comparison of absolute MHR values across studies additionally requires caution because the ratio is unit-dependent. In the present study, monocyte counts were expressed in 10^3^/µL and HDL in mg/dL, resulting in MHR values in the range of 0.002–0.025. Studies using 10^9^/L for monocytes and mmol/L for HDL report numerically different MHR values; reference ranges of approximately 0.175–0.709 in men and 0.135–0.511 in women have been reported using such conventions [16]. Consequently, raw MHR values and cut-offs should not be compared across studies without accounting for the underlying units. This issue is particularly important for future validation and argues for explicit standardization of MHR calculation and reporting.

Evidence from other neurological disorders provides biological context but should not be considered direct evidence for the role of MHR in MG. Higher MHR has been associated with baseline disability and relapse frequency in neuromyelitis optica spectrum disorder, with an independent association with severity in multivariable analysis (OR 7.90; 95% CI 1.08–57.82) [14]. MHR has also been reported to be elevated in the demyelinating subtype of Guillain–Barré syndrome [15] and in parkinsonian syndromes, including early stage disease [7]. These observations indicate that MHR may be responsive to inflammatory or metabolic alterations across several neurological conditions, but they also emphasize its lack of disease specificity.

The lipid findings in our cohort may be relevant to the MHR difference. Patients with MG had lower HDL, mildly higher triglycerides, and unchanged LDL and total cholesterol. A pattern of reduced HDL and increased triglycerides has also been described in autoimmune neurological diseases [29]. Conversely, in a cohort of 264 patients with MG, most lipid parameters were not associated with worsening at one year [30]. Thus, lipid alterations and prognostic information should be regarded as separate questions. In our data, lower HDL was not independently associated with MG after adjustment for BMI and hypertension (Table 7); the lipid differences may therefore reflect the metabolic profile of the patients rather than an MG-specific process, and the present findings do not establish that lipid parameters or MHR predict subsequent disease course.

The results for the other inflammatory indices provide context for the MHR finding. NLR was higher in patients with MG than in controls (2.58 vs. 1.81), consistent with previous reports. Yang et al. observed higher NLR in 172 patients with MG than in 207 healthy controls (1.94 vs. 1.60; *p* < 0.001) and reported an association with severe disease activity (OR 13.201; 95% CI 1.418–122.938) [12]. Huang et al. similarly reported higher NLR in patients than in controls (2.22 vs. 1.70; *p* < 0.001) [10], whereas Jin et al. reported higher NLR (2.33 vs. 1.72; adjusted *p* = 0.007) and an association with myasthenic crisis (OR 1.20; 95% CI 1.05–1.41) [8]. An association between NLR and disease severity has also been reported by Duan et al. [9]. Collectively, these studies support a relatively consistent association between NLR and MG, although the relationship with severity appears less uniform across populations (Table 10).

PLR was also higher in our MG group (147.1 vs. 119.7; *p* = 0.022; *q* = 0.027), but it had the lowest AUC among the evaluated ratios (0.603), and the difference was no longer significant in patients without diabetes or lipid-lowering therapy (*p* = 0.068) or in untreated patients (*p* = 0.359). Previous studies have likewise reported higher PLR in MG. Huang et al. observed values of 133.1 versus 110.9 (*p* < 0.001) and reported an association between PLR and respiratory failure (adjusted OR 1.026; *p* = 0.026; AUC 0.838 at a cut-off of 181.91) [10]. Jin et al. reported higher PLR (135.2 vs. 113.3; adjusted *p* = 0.007) and included PLR in LASSO-based models of MG status [8]. These findings concern different outcomes, however, and evidence regarding respiratory failure or other acute events should not be extrapolated to the ability of PLR to discriminate stable patients with MG from healthy individuals.

LMR was lower in our MG group (3.94 vs. 5.79; *p* < 0.001; *q* < 0.001) and had an AUC of 0.774. Huang et al. similarly reported lower LMR in MG (4.85 vs. 5.31; *p* = 0.002) and a weak negative correlation with MGFA classification (rs = −0.185; *p* = 0.047) [10]. Lower LMR has also been reported in severe disease [11] and in patients who did not respond to treatment (2.48 vs. 4.09; *p* < 0.001) [13]. In contrast, the LMR difference in the study by Jin et al. did not remain significant after Bonferroni correction (adjusted *p* = 0.380) [8]. In our cohort, the lower LMR occurred in the setting of a higher monocyte count, which also contributed to the higher MHR. Consistent with this shared component, MHR and LMR were inversely correlated (ρ = −0.264 within the MG group).

SIRI showed the highest numerical AUC in our cohort (0.779; adjusted OR 5.86), and the group difference remained significant after FDR correction (*q* < 0.001). Previous studies have primarily evaluated SIRI in relation to severity or outcome rather than case–control discrimination. Huang et al. reported an independent association with disease severity (OR 1.308; 95% CI 1.072–1.597; *p* = 0.008) and proposed a cut-off of 2.753 [11], whereas Chen et al. reported an association between SIRI and non-response to treatment at six months (OR 9.108; 95% CI 3.412–24.317), with an AUC of 0.841 at a cut-off of 1.62 [13]. These cut-offs should not be compared directly with our cut-off of 0.81 because the studies addressed different clinical outcomes and included different patient populations. Jin et al., moreover, found that SIRI did not remain significant after Bonferroni correction in either the group or severity comparisons [8]. Such heterogeneity reinforces the need to distinguish markers of disease presence from markers of severity and prognosis.

Importantly, none of the inflammatory ratios in our cohort was significantly associated with MGFA class or MG-ADL score; all absolute correlation coefficients with these scores were below 0.18, and no marker differed significantly between the mild and severe groups (all *p* > 0.15). These findings do not establish the absence of a severity relationship, particularly given the limited number of severe cases, but they indicate that no such association was detected in this cohort. This differs from studies reporting relationships of NLR, PLR, systemic immune-inflammation index (SII), or SIRI with disease severity [10,11,12]. However, Jin et al. found that associations of MHR (unadjusted p = 0.369; adjusted p = 1.000), LMR (adjusted *p* = 1.000), and SIRI (adjusted *p* = 0.153) with severity did not persist after Bonferroni correction, whereas NLR, neutrophil count, and SII retained a severity gradient [8]. The application of multiple-comparison correction is important when several correlated inflammatory indices are evaluated simultaneously, because nominally significant findings may otherwise reflect multiplicity.

Differences in patient selection may partly account for the heterogeneous severity findings. Our cohort included a relatively small severe subgroup, and samples were obtained both at outpatient visits and at hospital admissions. The median MG-ADL score was 6 (IQR 2–10), whereas a study reporting an association between SIRI and MG-ADL included substantially greater clinical separation, with median MG-ADL scores of 18 in severe cases and 6 in mild cases [11]. Restricted variation in disease activity may reduce the ability to detect biomarker–severity relationships. Concomitant infection is another potential contributor. In the same SIRI study, infection before admission was more frequent among patients with high SIRI (58.8% vs. 12.5%; *p* = 0.001) [11]. In our study, patients with active infection were excluded, although residual effects of subclinical infection and other inflammatory comorbidities cannot be excluded. Treatment may also be relevant: 55.0% of our patients were receiving corticosteroids or immunosuppressants at the time of sampling, and corticosteroids alter the distribution of circulating leukocytes, particularly lymphocytes [21]. However, marker values did not differ between treated and untreated patients, and the differences from controls in MHR, NLR, LMR, and SIRI, including the adjusted association of MHR, persisted in the 36 untreated patients (Table 9). Because treated patients more often had severe disease (MGFA class IV in 15 of 44 vs. 2 of 36), the effects of treatment and severity could not be separated in this cross-sectional sample.

These considerations are particularly important when interpreting the lack of association between MHR and severity. MHR may characterize a systemic inflammatory–metabolic phenotype that differs between MG and healthy controls without varying proportionally with contemporaneous clinical impairment. Alternatively, a true relationship with severity may have been obscured by the limited number of severe cases, treatment effects, restricted clinical variability, or residual confounding. Longitudinal measurements during exacerbation, treatment, and remission would be required to determine whether MHR changes with disease activity. The present cross-sectional data therefore do not establish MHR as a marker for monitoring treatment response or predicting exacerbation.

Because NLR, SIRI and LMR are built from the same neutrophil, lymphocyte and monocyte counts, concordant findings across these indices cannot be regarded as independent biological evidence. MHR differs from these indices in incorporating HDL cholesterol; however, in the component analysis its association with MG was explained by the monocyte count, and HDL did not contribute independently after adjustment for BMI and hypertension (Table 7). Adding MHR to the covariates improved discrimination (ΔAUC 0.070), but a model containing the monocyte count alone performed equally well. MHR did improve discrimination when added to a model containing CRP (ΔAUC 0.039). These increments are modest, were estimated in a single dataset, and require external validation.

In ROC analysis, SIRI and LMR had numerically higher AUC values than MHR, but the differences were not statistically significant by DeLong testing. This should not be interpreted as evidence that the markers have equivalent performance; rather, the analysis did not detect a statistically significant difference in discrimination in this sample. At a cut-off of 0.0075, MHR had a sensitivity of 65.8% and specificity of 72.4%. These values are insufficient to support MHR as a stand-alone diagnostic test. Moreover, the ROC analysis evaluated discrimination between an established MG cohort and healthy controls, which differs substantially from diagnostic performance in clinically relevant populations with suspected MG or alternative neuromuscular disorders. The diagnosis of MG remains based on the appropriate integration of clinical findings, electrophysiological testing, and serological evaluation. The present ROC results should therefore be regarded as exploratory measures of case–control discrimination rather than evidence of diagnostic utility.

The healthy-control design also affects interpretation of the multivariable analyses. Hypertension was strongly associated with MG status, most likely because controls were selected on the basis of health status rather than because of a disease-specific relationship. We therefore adjusted for hypertension and BMI to block the non-causal paths opened by this selection (Figure 2) [27]. Diabetes and lipid-lowering therapy could not be adjusted for, but restricting the analysis to patients without these conditions did not change the findings. Other unmeasured metabolic or inflammatory determinants may still contribute to the observed associations.

Several limitations should therefore be considered. First, the cross-sectional, single-center design prevents assessment of temporal relationships and limits causal inference and external validity. Moreover, 114 of the 194 screened patients were excluded, mainly because laboratory data were missing or because of comorbidities, and the excluded patients were younger than those analyzed, so the analyzed patients may not be fully representative of the patients with MG followed at our center. Second, the use of controls without known disease or regular medication, selected from check-up clinic records rather than by consecutive or random sampling and matched to the patients only at the group level, may accentuate differences in inflammatory and metabolic variables relative to those that would be observed in a clinically representative comparison group. Diabetes and lipid-lowering therapy were absent among controls and could not be incorporated into the multivariable models, leaving potential residual confounding despite sensitivity analyses. Third, BMI was missing for approximately 40% of participants. Missing values were handled by multiple imputation, and a complete-case analysis gave consistent results, but imputation relies on the assumption that data were missing at random. Fourth, retrospective sampling did not allow standardization of fasting duration or time of blood collection, which may affect lipid measurements and therefore MHR. Fifth, antibody results were unavailable for 43.0% of patients because testing had been performed at other centers before referral and the results could not be retrieved, preventing reliable evaluation of serological subgroups.

Sixth, only 17 patients were in the severe subgroup. The resulting restriction in the spectrum of disease activity limits the precision of analyses examining MGFA class and MG-ADL and may partly explain the absence of severity associations. In addition, although MGFA class and MG-ADL score were assessed at the sampling visit, samples were obtained both at outpatient visits and at hospital admissions, including exacerbations, and the setting could not be reliably determined for each patient from the records; the markers therefore could not be analyzed by sampling setting. Furthermore, more than half of the patients were receiving corticosteroids or immunosuppressants when sampled; although the findings were consistent in untreated patients, the effects of treatment dose and duration could not be evaluated. In addition, four patients with CRP above 10 mg/L were retained because the elevation was attributed to exacerbation or recent thymectomy, whereas controls with such values were excluded; this asymmetry could inflate between-group differences, although excluding these patients did not change the results. Seventh, NLR showed a non-linear association with MG status, so its OR should be interpreted with caution. All models also showed modest optimism on internal validation (calibration slopes 0.84–0.86) and require external validation. Eighth, the ROC cut-offs were derived and evaluated within the same dataset and may therefore overestimate performance. They should be considered exploratory until externally validated. Ninth, the substantial mathematical and biological overlap among MHR and the CBC-derived inflammatory indices raises the possibility of collinearity and redundant information, particularly when several ratios are entered into model-selection procedures. Finally, measurements were obtained at a single time point, precluding evaluation of within-patient changes with exacerbation, treatment, or remission. The discordance between our MHR result and that reported by Jin et al. [8] further underscores the need to establish reproducibility across populations and analytical settings.

Prospective multicenter studies should therefore evaluate MHR using standardized units and sampling conditions, more representative control groups, and adequate adjustment for BMI, cardiovascular and metabolic comorbidities, infection, lipid-lowering therapy, and immunotherapy. Longitudinal sampling would help determine whether MHR is stable within individuals or changes with MG activity and treatment. External validation of ROC thresholds and multivariable models, including assessment of both discrimination and calibration, will be necessary before considering clinical applications. Studies with larger numbers of patients across the full spectrum of MG severity and with more complete antibody characterization may also clarify whether MHR differs across clinically or biologically defined subgroups.

## 5. Conclusions

MHR was higher in patients with MG than in healthy controls in this cohort and remained associated with MG status after adjustment for age, sex, smoking, BMI, and hypertension. Its discriminative information was carried mainly by the monocyte count, and HDL did not contribute independently after adjustment for BMI and hypertension, although MHR improved discrimination beyond CRP. However, MHR was not associated with MG severity in this cohort, and the present findings do not establish a diagnostic, prognostic, or monitoring role. The discrepancy with previously published MHR data, together with the unit-dependent nature of the ratio and the limitations of internally derived ROC thresholds, makes independent prospective validation essential before its clinical relevance in MG can be determined.

## Figures and Tables

**Figure 1:**
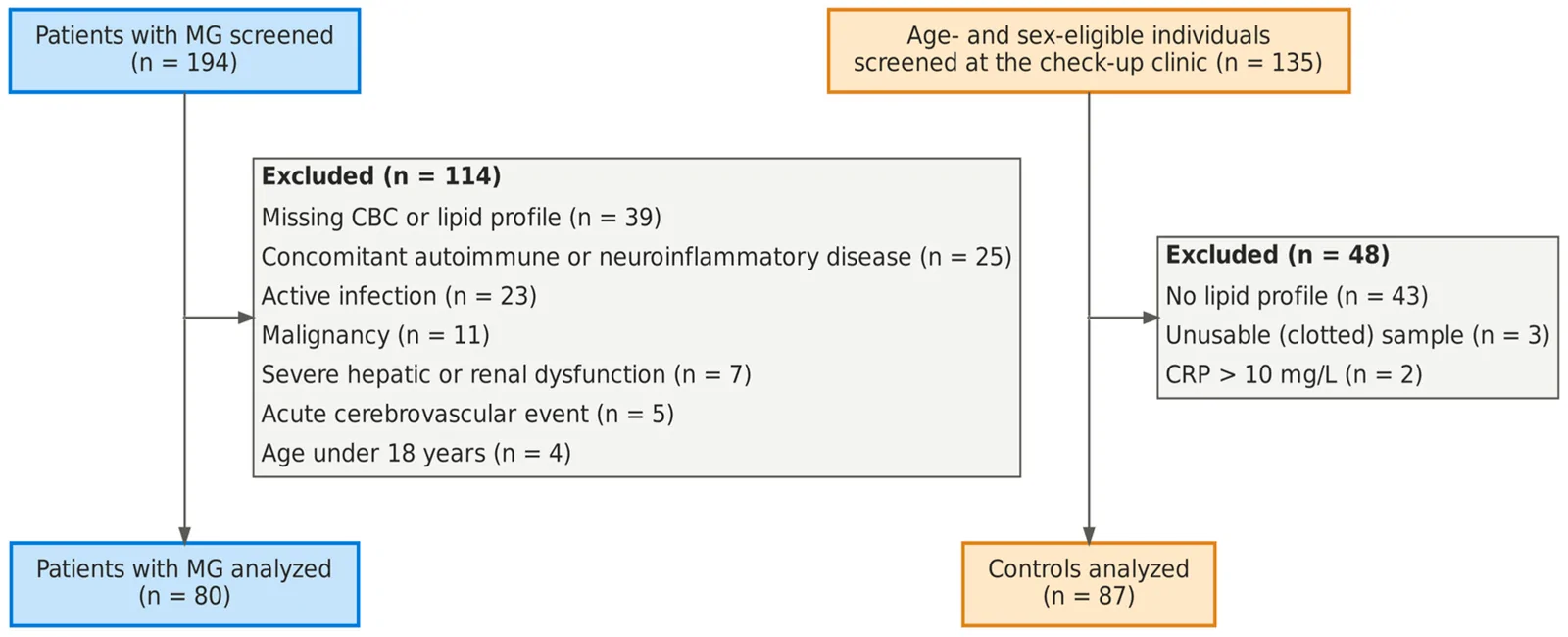
Flow diagram of participant selection. Each excluded individual is listed under a single reason. CBC, complete blood count; CRP, C-reactive protein; MG, myasthenia gravis.

**Figure 2:**
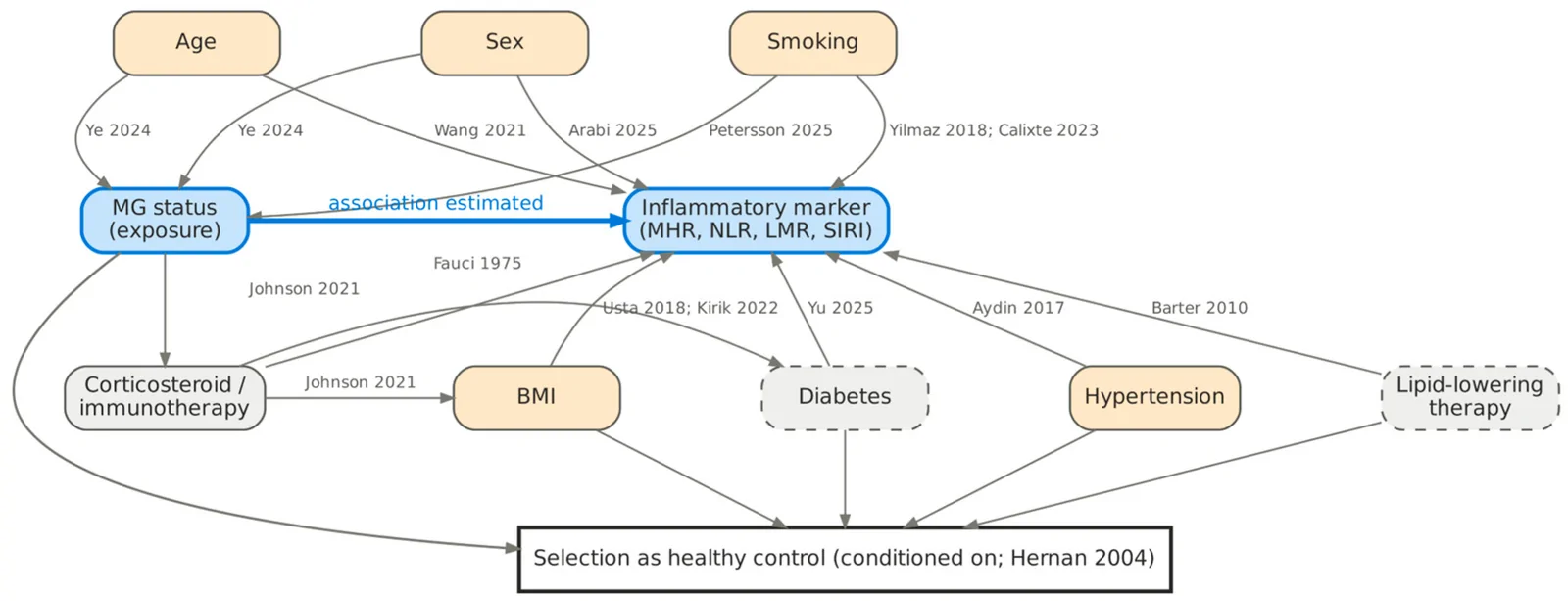
Directed acyclic graph used to select covariates. Blue boxes mark the exposure (MG status) and the outcome of interest (inflammatory markers); the thick blue arrow is the association estimated. Orange boxes are the adjustment variables: age, sex, and smoking (Model 1) and, additionally, BMI and hypertension (Model 2). Diabetes and lipid-lowering therapy (grey, dashed) could not be adjusted for because no control had either condition and were addressed by restriction. Corticosteroid or other immunotherapy (grey) is a consequence of MG that may affect the markers and was not adjusted for. The bottom box represents the selection of controls on the basis of health status; conditioning on this selection opens non-causal paths through hypertension, BMI, diabetes and lipid-lowering therapy. Edge labels give the first author and year of the supporting reference: Ye 2024 [17], Petersson 2025 [18], Wang 2021 [28], Arabi 2025 [16], Yılmaz 2018 [19], Calixte 2023 [20], Fauci 1975 [21], Johnson 2021 [22], Usta 2018 [5], Kirik 2022 [23], Aydin 2017 [24], Yu 2025 [25], Barter 2010 [26], Hernán 2004 [27].

**Figure 3:**
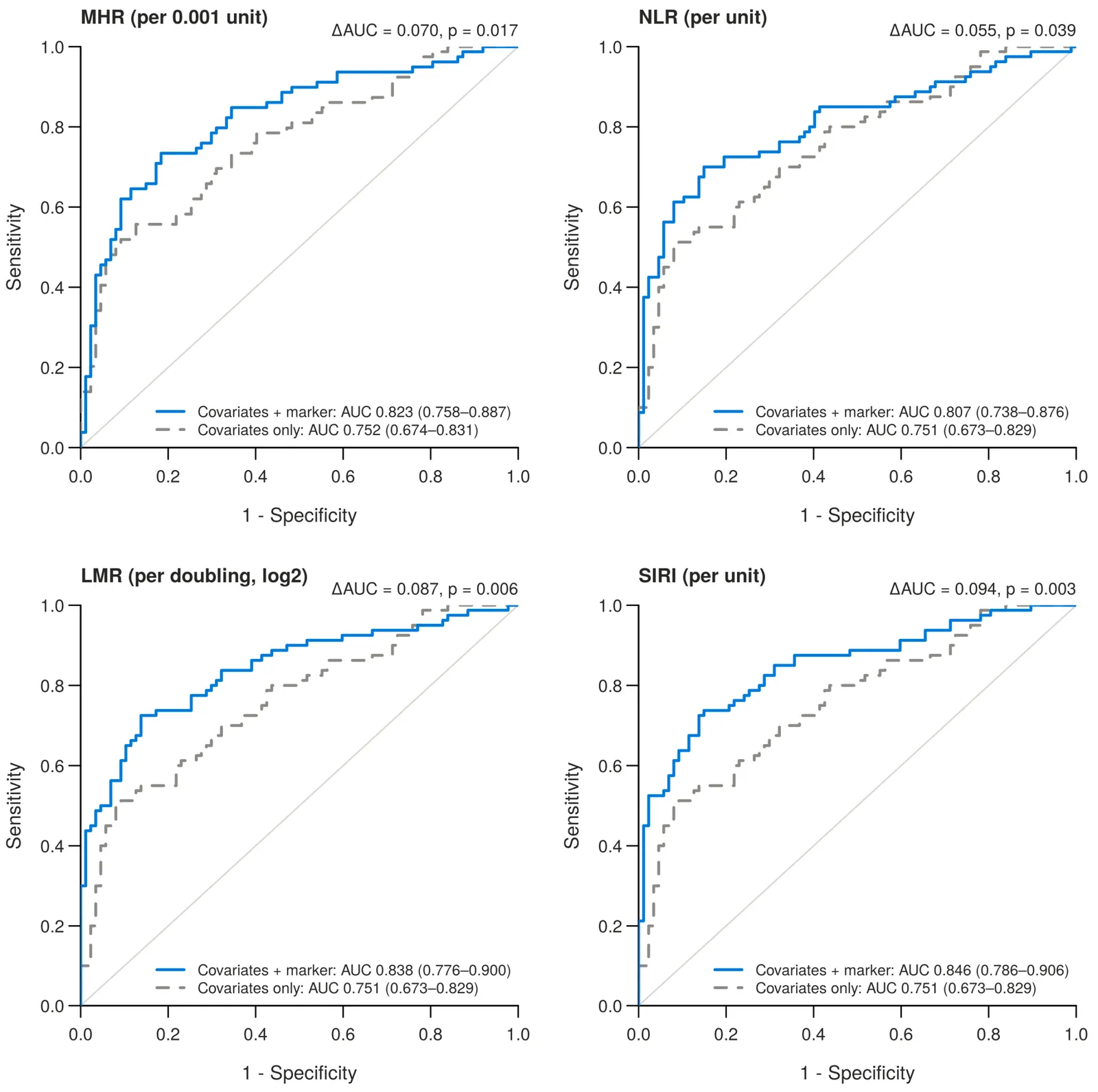
ROC curves of the multivariable models. Solid blue lines show Model 2 (age, sex, smoking, BMI, hypertension, and the marker); dashed grey lines show the same covariates without the marker. Curves use predicted probabilities averaged over 40 imputed datasets; AUCs, 95% Cis, and ΔAUC *p*-values (paired DeLong test) are pooled across imputations with Rubin’s rules. LMR is modeled on the log2 scale.

**Figure 4:**
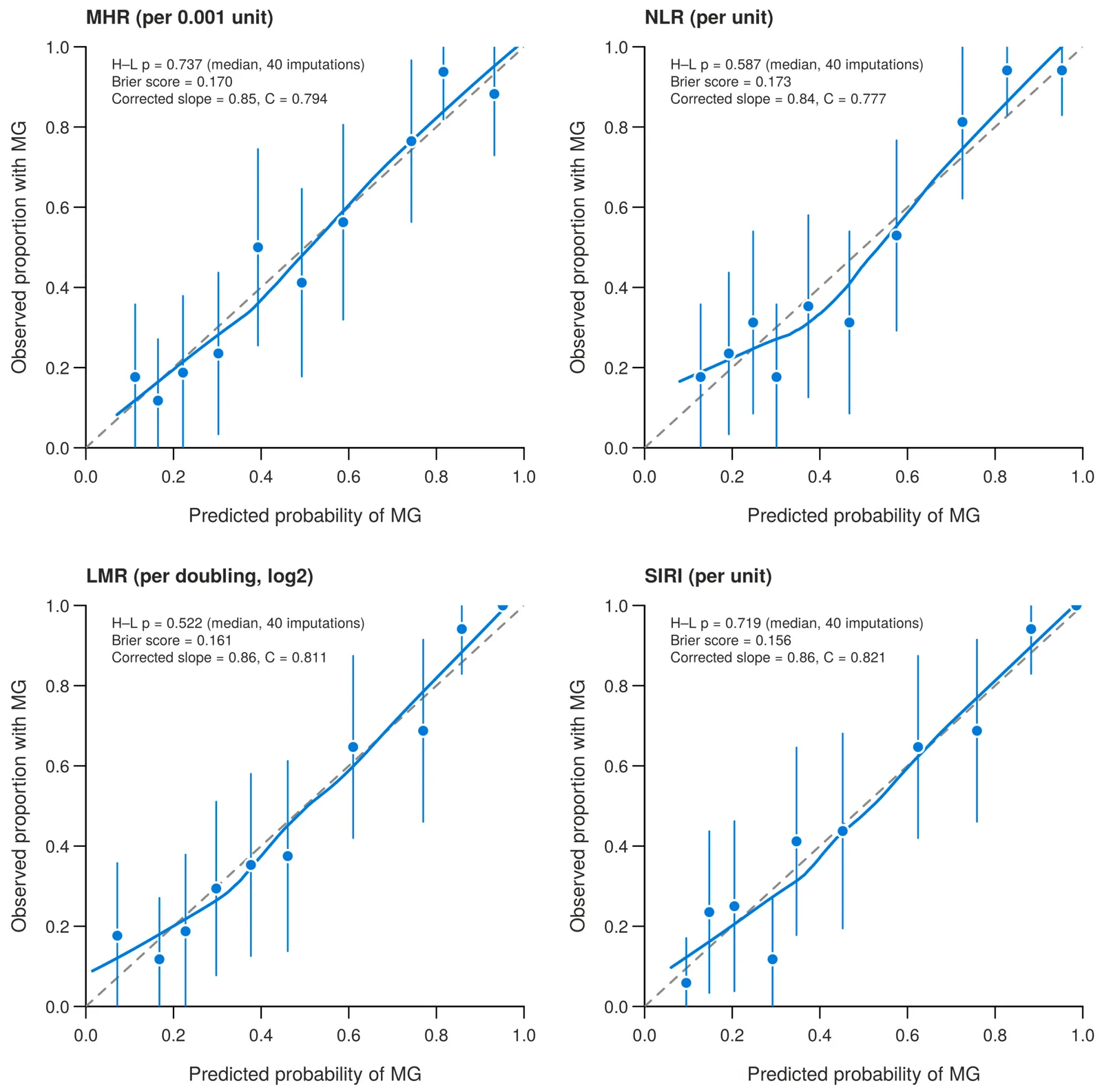
Calibration of the multivariable models (Model 2). Points show the observed proportion of patients with MG within deciles of predicted probability (vertical lines, 95% CI); the blue line is a lowess smoother and the dashed line marks perfect calibration. H–L, Hosmer–Lemeshow test (median *p* across 40 imputations). Calibration slope and C-statistic are optimism-corrected by bootstrap (200 resamples per imputation).

**Table 1:** Demographic characteristics.

Variable	Controls (*n* = 87)	MG (*n* = 80)	Test	*p*
Age, years, median (IQR)	62 (44–78)	64 (53–74)	*Z* = −0.675	0.500
Sex, *n* (%)			χ^2^ = 0.033	0.856
Female	51 (58.6)	48 (60.0)		
Male	36 (41.4)	32 (40.0)		
Smoking, *n* (%)			χ^2^ = 3.60	0.165
Never	56 (64.4)	40 (50.0)		
Former	15 (17.2)	18 (22.5)		
Current	16 (18.4)	22 (27.5)		
BMI, kg/m^2^, median (IQR)	25.6 (23.4–29.4)	29.4 (25.4–32.5)	Z = −2.921	0.003
Hypertension, *n* (%)	15 (17.2)	44 (55.0)	χ^2^ = 26.01	<0.001
Diabetes, *n* (%)	0 (0)	18 (22.5)	—	—
Lipid-lowering therapy, *n* (%)	0 (0)	12 (15.0)	—	—

BMI, body mass index; IQR, interquartile range; MG, myasthenia gravis; Z, Mann–Whitney U test (normal approximation); χ^2^, Pearson chi-square test. BMI data were available for 50 controls and 51 patients with MG. Because diabetes and lipid-lowering therapy had a frequency of zero in the control group, no statistical comparison was performed.

**Table 2:** Clinical characteristics of patients with MG (*n* = 80).

Characteristic	*n* (%)/Median (IQR)	Data Available, *n*
Disease duration, months	53.9 (18.9–104.3)	80
MG type		80
Generalized	63 (78.8)	
Ocular	17 (21.3)	
Antibody status		79
AChR-positive	25 (31.6)	
MuSK-positive	0 (0.0)	
Seronegative	20 (25.3)	
Unknown (tested elsewhere, result unavailable)	34 (43.0)	
MGFA class		80
I	21 (26.3)	
II	20 (25.0)	
III	22 (27.5)	
IV	17 (21.3)	
MGFA group		80
Mild (I–III)	63 (78.8)	
Severe (IV–V)	17 (21.3)	
MG-ADL score	6 (2–10)	80
History of myasthenic crisis	41 (51.3)	80
Thymectomy	18 (22.8)	79
Thymic histopathology (thymectomized patients)		18
Thymoma	13 (72.2)	
Thymic hyperplasia	5 (27.8)	
Refractory disease	8 (10.1)	79
Treatment at the time of sampling		80
Pyridostigmine	73 (91.3)	
Corticosteroids	14 (17.5)	
Azathioprine	30 (37.5)	
Rituximab or eculizumab	2 (2.5)	
Any corticosteroid or immunosuppressant	44 (55.0)	
None of the above (pyridostigmine only or untreated)	36 (45.0)	

AChR, acetylcholine receptor; IQR, interquartile range; MG-ADL, Myasthenia Gravis Activities of Daily Living score; MGFA, Myasthenia Gravis Foundation of America classification; MuSK, muscle-specific tyrosine kinase. Percentages were calculated based on the number of cases with available data. Antibody status was classified as unknown when testing had been performed at another center before referral, and the result could not be retrieved. Thymic histopathology is shown for patients who underwent thymectomy. Treatment at the time of sampling refers to medications in use on the date of blood sampling; the drug categories are not mutually exclusive.

**Table 3:** Laboratory parameters and inflammatory ratios.

Parameter	Controls (*n* = 87)	MG (*n* = 74–80)	Z	*p*	*q*
WBC, ×10^3^/µL	6.66 (5.46–7.70)	7.41 (6.12–9.02)	−2.903	0.004	0.006
Neutrophils, ×10^3^/µL	3.73 (3.08–4.41)	4.71 (3.54–6.13)	−3.918	<0.001	<0.001
Lymphocytes, ×10^3^/µL	2.15 (1.77–2.43)	1.71 (1.30–2.24)	−3.436	<0.001	0.001
Monocytes, ×10^3^/µL	0.34 (0.30–0.41)	0.45 (0.38–0.60)	−4.686	<0.001	<0.001
Platelets, ×10^3^/µL	261 (222–298)	255 (223–285)	−0.650	0.515	0.558
HDL, mg/dL	56 (49–66)	50 (43–58)	−2.927	0.003	0.006
LDL, mg/dL	111 (90–136)	112 (88–140)	−0.641	0.521	0.558
Total cholesterol, mg/dL	193 (163–220)	190 (160–224)	−0.215	0.830	0.830
Triglycerides, mg/dL	108 (68–142)	118 (96–164)	−2.668	0.008	0.010
CRP, mg/L	1.20 (0.95–3.20)	3.00 (2.00–4.00)	−3.984	<0.001	<0.001
MHR	0.0058 (0.0046–0.0085)	0.0091 (0.0062–0.0126)	−4.446	<0.001	<0.001
NLR	1.81 (1.52–2.09)	2.58 (1.72–3.82)	−4.628	<0.001	<0.001
PLR	119.7 (95.7–150.9)	147.1 (99.7–204.0)	−2.291	0.022	0.027
LMR	5.79 (4.55–7.46)	3.94 (2.99–5.04)	−6.109	<0.001	<0.001
SIRI	0.62 (0.47–0.82)	1.12 (0.75–2.24)	−6.212	<0.001	<0.001

Values are median (interquartile range). Mann–Whitney U test. *q*, Benjamini–Hochberg false discovery rate–adjusted *p*-value; the family of tests was prespecified as all 15 between-group comparisons in this table. MG, *n* = 80 except HDL, triglycerides and MHR (*n* = 79), LDL (*n* = 78) and CRP (*n* = 74). CRP, C-reactive protein; HDL, high-density lipoprotein; LDL, low-density lipoprotein; LMR, lymphocyte-to-monocyte ratio; MHR, monocyte-to-HDL cholesterol ratio; NLR, neutrophil-to-lymphocyte ratio; PLR, platelet-to-lymphocyte ratio; SIRI, systemic inflammation response index; WBC, white blood cell count.

**Table 4:** Results of ROC analysis.

Marker	AUC	95% CI	*p*	Cut-Off	Sensitivity (%)	Specificity (%)
SIRI	0.779	0.707–0.850	<0.001	>0.81	72.5	72.4
LMR	0.774	0.703–0.845	<0.001	≤4.42	68.8	80.5
NLR	0.708	0.624–0.791	<0.001	>2.30	60.0	85.1
MHR	0.700	0.619–0.781	<0.001	>0.0075	65.8	72.4
PLR	0.603	0.513–0.692	0.022	>199.3	30.0	96.6

AUC, area under the curve; CI, confidence interval. Cut-off values were determined using the Youden index; for LMR, values at or below the cut-off indicate MG. In paired DeLong tests (*n* = 166), the AUC of MHR did not differ significantly from those of SIRI (*p* = 0.066), LMR (*p* = 0.103), NLR (*p* = 0.915) or PLR (*p* = 0.136).

**Table 5:** Association of each inflammatory marker with MG status in multivariable logistic regression models.

Model	MHR (per 0.001 Unit)	NLR (per Unit)	LMR (per Doubling)	SIRI (per Unit)
Crude	1.20 (1.10–1.30) *p* < 0.001	2.13 (1.48–3.06) *p* < 0.001	0.17 (0.09–0.33) *p* < 0.001	6.77 (3.18–14.43) *p* < 0.001
Model 1	1.24 (1.12–1.36) *p* < 0.001	2.12 (1.47–3.06) *p* < 0.001	0.17 (0.08–0.33) *p* < 0.001	6.62 (3.11–14.10) *p* < 0.001
Model 2 (primary)	1.23 (1.11–1.38) *p* < 0.001	1.85 (1.28–2.68) *p* = 0.001	0.19 (0.09–0.38) *p* < 0.001	5.86 (2.63–13.07) *p* < 0.001
Model 2, complete-case	1.22 (1.04–1.42) *p* = 0.012	1.38 (0.99–1.93) *p* = 0.059	0.22 (0.07–0.69) *p* = 0.009	3.47 (1.42–8.47) *p* = 0.006
Model 2, restricted	1.20 (1.07–1.35) *p* = 0.002	1.70 (1.22–2.37) *p* = 0.002	0.21 (0.10–0.44) *p* < 0.001	4.70 (2.19–10.07) *p* < 0.001

Values are odds ratios (95% CI) and *p*-values. Model 1: adjusted for age, sex, and smoking (minimal adjustment set from the DAG; Figure 2). Model 2 (primary): Model 1 + BMI + hypertension; missing BMI multiply imputed (40 datasets); *n* = 167 (166 for MHR). Complete-case: participants with recorded BMI (*n* = 101). Restricted: Model 2 after excluding patients with MG who had diabetes or used lipid-lowering therapy (*n* = 140; 139 for MHR). LMR was modeled on the log2 scale. In Model 2, hypertension was associated with MG in all models (OR 6.39–9.00; all *p* < 0.001), whereas BMI was not (OR 1.01–1.03; all *p* > 0.5). BMI, body mass index; CI, confidence interval; DAG, directed acyclic graph; OR, odds ratio.

**Table 6:** Discrimination, fit, and calibration of the multivariable models (Model 2).

Statistic	MHR	NLR	LMR (log2)	SIRI
AUC, model (95% CI)	0.823 (0.758–0.887)	0.807 (0.738–0.876)	0.838 (0.776–0.900)	0.846 (0.786–0.906)
AUC, covariates only	0.752	0.751	0.751	0.751
ΔAUC (95% CI)	0.070 (0.012–0.128)	0.055 (0.003–0.108)	0.087 (0.025–0.148)	0.094 (0.032–0.156)
*p* for ΔAUC	0.017	0.039	0.006	0.003
Optimism-corrected C	0.794	0.777	0.811	0.821
Calibration slope (corrected)	0.85	0.84	0.86	0.86
Hosmer–Lemeshow *p* (median)	0.737	0.587	0.522	0.719
Nagelkerke R^2^	0.388	0.383	0.437	0.470
Cox–Snell R^2^	0.291	0.287	0.327	0.352
−2 log-likelihood	172.7	174.6	165.0	158.7
AIC	188.7	190.6	181.0	174.7
Brier score	0.170	0.173	0.161	0.156
Correctly classified (%)	76.1	75.6	77.8	78.5

AUCs and ΔAUC were pooled across 40 imputed datasets (DeLong variance and Rubin’s rules); *p* for ΔAUC from the paired DeLong test comparing Model 2 with the covariates-only model (age, sex, smoking, BMI, and hypertension). Optimism-corrected C and calibration slope were obtained from 200 bootstrap resamples per imputation. ΔAUC was pooled directly across imputations and may differ by 0.001 from the difference between the pooled AUCs. Hosmer–Lemeshow test with 10 groups; median *p* across imputations. The likelihood-ratio test for adding the marker was *p* < 0.001 in every model. Complete-case results are given in Supplementary Table S1. AIC, Akaike information criterion; AUC, area under the curve.

**Table 7:** Contribution of the MHR components (monocyte count and HDL cholesterol) to the multivariable model.

Model	Added Term, OR (95% CI)	*p*	AUC (95% CI)	ΔAUC (*p*)	AIC
A. Covariates only	—	—	0.752 (0.674–0.831)	Reference	207.2
B. A + MHR	MHR 1.23 (1.11–1.38)	<0.001	0.823 (0.758–0.887)	vs. A: 0.070 (0.017)	188.7
C. A + monocyte count	Monocyte 1.81 (1.35–2.43)	<0.001	0.826 (0.763–0.890)	vs. A: 0.074 (0.016); vs. B: 0.004 (0.753)	185.2
D. A + HDL	HDL 0.79 (0.59–1.06)	0.118	0.767 (0.694–0.840)	vs. A: 0.015 (0.354)	206.4
E. A + monocyte count + HDL	Monocyte 1.78 (1.33–2.40); HDL 0.90 (0.67–1.23)	<0.001; 0.510	0.827 (0.764–0.891)	vs. B: 0.005 (0.610)	186.7
F. E + MHR	MHR 0.93 (0.61–1.40)	0.717	0.826 (0.763–0.890)	vs. E: LR test *p* = 0.726	188.6
G. A + log CRP	—	—	0.811 (0.746–0.877)	Reference	191.5
H. G + MHR	MHR 1.21 (1.08–1.35)	0.001	0.850 (0.791–0.909)	vs. G: 0.039 (0.044)	178.4

Covariates (model A): age, sex, smoking, BMI, and hypertension (Model 2 covariates). All estimates were pooled across 40 imputed datasets (*n* = 166). ORs are expressed per 0.001 unit for MHR, per 0.1 × 10^3^/µL for the monocyte count, and per 10 mg/dL for HDL cholesterol. ΔAUC values are differences from the indicated model (paired DeLong test, Rubin’s rules). LR, likelihood-ratio test (median *p* across imputations). Test of the ratio constraint (log MHR vs. log monocyte count and log HDL as separate terms): median *p* = 0.105. AIC, Akaike information criterion; AUC, area under the curve; CRP, C-reactive protein; HDL, high-density lipoprotein; MHR, monocyte-to-HDL cholesterol ratio; OR, odds ratio.

**Table 8:** Correlations of inflammatory ratios with indicators of clinical severity (MG group and Spearman’s ρ).

Marker	MGFA Class	MG-ADL Score	Disease Duration	MGFA Mild vs. Severe, *p*
MHR	−0.175 (0.123)	−0.176 (0.121)	0.050 (0.660)	0.154
NLR	−0.099 (0.383)	−0.037 (0.745)	0.141 (0.213)	0.773
PLR	0.080 (0.481)	0.036 (0.754)	0.211 (0.060)	0.182
LMR	0.057 (0.613)	0.084 (0.457)	−0.097 (0.393)	0.764
SIRI	−0.077 (0.497)	−0.048 (0.670)	0.119 (0.292)	0.842

**Table 9:** Sensitivity analyses.

Subgroup of Patients with MG	Marker	Controls (*n* = 87)	Patients with MG	*p*
Without diabetes or lipid-lowering therapy (*n* = 53)	MHR	0.0058 (0.0046–0.0085)	0.0079 (0.0057–0.0122)	<0.001
	NLR	1.81 (1.52–2.09)	2.20 (1.52–4.29)	0.015
	PLR	119.7 (95.7–150.9)	141.9 (98.8–202.2)	0.068
	LMR	5.79 (4.55–7.46)	4.04 (3.27–5.62)	<0.001
	SIRI	0.62 (0.47–0.82)	1.00 (0.64–2.06)	<0.001
No corticosteroid or immunosuppressant at sampling (*n* = 36)	MHR	0.0058 (0.0046–0.0085)	0.0095 (0.0073–0.0127)	<0.001
	NLR	1.81 (1.52–2.09)	2.52 (1.64–3.35)	0.005
	PLR	119.7 (95.7–150.9)	128.0 (102.6–174.4)	0.359
	LMR	5.79 (4.55–7.46)	4.00 (3.50–5.04)	<0.001
	SIRI	0.62 (0.47–0.82)	1.10 (0.69–1.70)	<0.001
CRP ≤ 10 mg/L (*n* = 76)	MHR	0.0058 (0.0046–0.0085)	0.0091 (0.0064–0.0126)	<0.001
	NLR	1.81 (1.52–2.09)	2.58 (1.72–3.77)	<0.001
	PLR	119.7 (95.7–150.9)	147.1 (102.1–202.6)	0.019
	LMR	5.79 (4.55–7.46)	3.94 (2.99–4.88)	<0.001
	SIRI	0.62 (0.47–0.82)	1.12 (0.75–2.22)	<0.001

**Table 10:** Comparison of the present findings with studies of CBC-derived markers in MG.

Study	MG/Control, *n*	Markers Examined	Differences vs. Controls	Association with Severity
Present study	80/87	MHR, NLR, PLR, LMR, SIRI	MHR, NLR, PLR, SIRI higher; LMR lower	None (all *p* > 0.12)
Jin et al., 2026 [8]	134/58	NLR, PLR, SII, SIRI, MHR, LMR	NLR, PLR, SII (MHR, LMR, SIRI not different *)	NLR: yes; MHR, LMR, SIRI: no *
Duan et al., 2022 [9]	128/116	NLR	—	Yes
Huang X et al., 2023 [10]	117/120	NLR, PLR, LMR, SII	NLR, PLR, SII higher; LMR lower	NLR, PLR, SII positive; LMR negative (r_s_ = −0.185)
Huang S et al., 2024 [11]	143/—	SIRI, NLR, PLR, LMR, SII	—	SIRI independent (OR 1.308); *r* = 0.232 with MG-ADL
Yang et al., 2017 [12]	172/207	NLR	NLR (1.94 vs. 1.60)	Yes (OR 13.201)
Chen et al., 2025 [13]	260/—	SIRI, PNI, NLR, PLR, LMR	—	SIRI associated with treatment non-response (OR 9.108)

OR, odds ratio; PNI, prognostic nutritional index; SII, systemic immune-inflammation index. Numbers in brackets correspond to reference numbers. For studies that examined patient groups only, the control-comparison column was left blank. In the study by Huang S et al. [11], 171 patients were screened, and 143 were analyzed after exclusions. * In the series of Jin et al. [8], although LMR and SIRI appeared to differ in the unadjusted analysis, they lost significance after Bonferroni correction; MHR was not significant even before correction (MG 0.29 vs. control 0.31; *p* = 0.288).

## Data Availability

The datasets generated and analyzed during the current study are not publicly available due to privacy and ethical restrictions but are available from the corresponding author upon reasonable request.

## Supplementary Materials

The following supporting information can be downloaded at: https://www.mdpi.com/article/10.3390/jcm15218738/s1, Table S1: Fit and discrimination of the multivariable models in the complete-case analysis (Model 2; participants with recorded BMI); Table S2: Tests of linearity of the inflammatory markers in the logit (Model 2).

## Abbreviations

The following abbreviations are used in this manuscript:

AChR	acetylcholine receptor
AIC	Akaike information criterion
AUC	area under the curve
BMI	body mass index
CBC	complete blood count
CI	confidence interval
CRP	C-reactive protein
DAG	directed acyclic graph
FDR	false discovery rate
HDL	high-density lipoprotein
IQR	interquartile range
LDL	low-density lipoprotein
LMR	lymphocyte-to-monocyte ratio
LRP4	low-density lipoprotein receptor-related protein 4
MG	myasthenia gravis
MG-ADL	Myasthenia Gravis Activities of Daily Living
MGFA	Myasthenia Gravis Foundation of America
MHR	monocyte-to-HDL cholesterol ratio
MuSK	muscle-specific tyrosine kinase
NLR	neutrophil-to-lymphocyte ratio
OR	odds ratio
PLR	platelet-to-lymphocyte ratio
PNI	prognostic nutritional index
ROC	receiver operating characteristic
SII	systemic immune-inflammation index
SIRI	systemic inflammation response index
