# Supplementary material for: Monocyte-to-HDL Cholesterol Ratio and Complete Blood Count-Derived Inflammatory Markers in Myasthenia Gravis: A Cross-Sectional Case–Control Study
Source: J Clin Med. 2026 Nov 9;15(21):8738. doi: 10.3390/jcm15218738 (PMC13764055; doi:10.3390/jcm15218738)
Supplement: Supplementary file 1 [file jcm-15-08738-s001.zip › jcm-4659941-supplementary.pdf]

# Monocyte-to-HDL Cholesterol Ratio and Other Complete Blood Count–Derived Inflammatory Markers in Myasthenia Gravis: A Cross-Sectional Case–Control Study

## Supplementary Materials

**Table S1.** Fit and discrimination of the multivariable models in the complete-case analysis (Model 2; participants with recorded BMI).

| **Statistic** | **MHR** | **NLR** | **LMR (log2)** | **SIRI** |
|---|---|---|---|---|
| *n* | 101 | 101 | 101 | 101 |
| AUC, model (95% CI) | 0.905 (0.845–0.964) | 0.903 (0.843–0.963) | 0.918 (0.865–0.972) | 0.919 (0.863–0.974) |
| AUC, covariates only | 0.876 | 0.876 | 0.876 | 0.876 |
| $p$ for ΔAUC (DeLong) | 0.216 | 0.213 | 0.089 | 0.105 |
| Omnibus $\chi^2$ (df) | 61.6 (7); $p < 0.001$ | 60.5 (7); $p < 0.001$ | 62.7 (7); $p < 0.001$ | 65.2 (7); $p < 0.001$ |
| −2 log-likelihood | 78.4 | 79.5 | 77.3 | 74.8 |
| Nagelkerke $R^2$ | 0.609 | 0.601 | 0.617 | 0.634 |
| Cox–Snell $R^2$ | 0.457 | 0.450 | 0.463 | 0.475 |
| Hosmer–Lemeshow $\chi^2$ (df) | 3.15 (8); $p = 0.925$ | 15.18 (8); $p = 0.056$ | 9.89 (8); $p = 0.273$ | 13.16 (8); $p = 0.106$ |
| Brier score | 0.124 | 0.124 | 0.124 | 0.113 |
| Correctly classified (%) | 82.2 | 80.2 | 79.2 | 83.2 |

Model 2: age, sex, smoking, BMI, hypertension and the marker, fitted in the 101 participants with recorded BMI (50 controls, 51 patients with MG). $p$ for ΔAUC from the paired DeLong test comparing Model 2 with the covariates-only model. The covariates-only model had a higher AUC in this subgroup than in the full imputed sample (0.752), because controls with recorded BMI were younger than those without. AUC, area under the curve; BMI, body mass index; LMR, lymphocyte-to-monocyte ratio; MHR, monocyte-to-HDL cholesterol ratio; NLR, neutrophil-to-lymphocyte ratio; SIRI, systemic inflammation response index.

**Table S2.** Tests of linearity of the inflammatory markers in the logit (Model 2).

| **Marker** | **Scale in the model** | ***p* for non-linearity, median (range)** | **Interpretation** |
|---|---|---|---|
| MHR | per 0.001 unit | 0.639 (0.540–0.809) | Linear |
| NLR | per 1 unit | 0.005 (0.004–0.009) | Non-linear |
| LMR | per doubling (log2) | 0.345 (0.274–0.393) | Linear |
| SIRI | per 1 unit | 0.918 (0.804–0.946) | Linear |

Likelihood-ratio test comparing a linear term with a natural cubic spline (3 degrees of freedom) for the marker in Model 2, in each of the 40 imputed datasets; the median and range of the $p$ values are shown. On its original scale, LMR departed from linearity ($p = 0.002$ in Model 1) and was therefore modeled on the log2 scale. NLR remained non-linear after log transformation and was kept on its original scale.
